# Impact of general anesthesia on feline aqueous tear production and the feline corneal epithelium

**DOI:** 10.1177/1098612X251386135

**Published:** 2025-09-29

**Authors:** Kaitlyn N Haubrich, Marina L Leis, Shayna M Levitt, Sarah E Parker, Lynne S Sandmeyer

**Affiliations:** 1 Department of Small Animal Clinical Sciences, Western College of Veterinary Medicine, Saskatoon, SK, Canada; 2 Department of Large Animal Clinical Sciences, Western College of Veterinary Medicine, Saskatoon, SK, Canada

**Keywords:** Tear production, corneal ulceration, general anesthesia, ocular lubrication

## Abstract

**Objectives:**

The aim of the present study was to identify the prevalence of corneal injury in cats undergoing general anesthesia (GA) while receiving prophylactic ocular lubrication, identify risk factors for corneal injury and quantify the effect of GA on tear production in cats.

**Methods:**

A total of 42 cats undergoing GA for non-ophthalmic procedures were included. Before GA, an ocular examination including a Schirmer tear test-1 (STT-1) and fluorescein stain (FS) was performed. Prophylactic lubrication was administered at the time of anesthetic induction and repeated every 15 mins until extubation. At 1 h after extubation, STT-1 and FS were performed and repeated hourly for 4 h. A Shapiro–Wilk test and paired *t*-test compared STT-1 results before and after GA. Logistic regression was used to analyze corneal injury and possible risk factors for corneal injury.

**Results:**

No cats developed FS uptake consistent with corneal ulceration. In total, 14 cats and 23 (27.4%) eyes developed corneal erosion at all time points. There was a significant decrease in tear production at all four time points after GA. Pre-medication opioid choice and corneal exposure were identified as significant risk factors for corneal injury.

**Conclusions and relevance:**

Corneal ulceration did not develop after GA in this study. There was a significant decrease in tear production in cats for at least 4 h after GA. Cats appear to have a higher prevalence of corneal injury after GA compared with dogs. Frequent eye lubrication is recommended for feline patients during and after GA.

## Introduction

Corneal erosion or ulceration after general anesthesia (GA) has been well documented.^1–3^ The relationship between corneal injury and GA is likely multifactorial and not fully understood. Reported risk factors in dogs and humans include lagophthalmos and relaxation of the orbiculus oculi muscles, diminished protective reflexes, the inability to blink and/or close the eyes, conformational differences such as brachycephalic dogs, anesthetic drug class, generalized stress and stress specific to hospitalization, eye position during surgery, duration of anesthesia, direct trauma, chemical injury, reduced aqueous tear production, and overall corneal exposure and drying.^1,2,4–7^

Anesthesia is known to reduce tear production in dogs and cats. Significant decreases in tear production have been documented for up to 24 h after GA in dogs and up to 18 h after GA in cats.^8,9^ Wolfran et al^10^ reported that common sedation agents, such as dexmedetomidine and methadone, temporarily decrease aqueous tear production in healthy cats when used alone or in combination. When the alpha-2 agonist was reversed, there was a significant improvement in aqueous tear production. It is theorized that aqueous tear production in healthy cats can be impacted by stress and the sympathetic autonomic nervous system, with conflicting evidence in the current literature.^11–13^ Other causes for decreased tear production associated with GA include inhalational anesthetics, anticholinergics and length of anesthesia.^14^

The prevalence of corneal epithelial defects in humans after GA is reported to be up to 10% in unprotected eyes and as little as 0.17% in prophylactically lubricated eyes.^15,16^ A retrospective study of 14 dogs reported a prevalence of GA-associated corneal ulceration in 1.9% with prophylactic lubrication.^14^ A prospective study of dogs showed that prophylactic ocular lubrication applied at the beginning, and every 2–4 h during GA, was associated with a low prevalence of corneal ulceration (0.5%); however, corneal erosion was noted in 18.6% of eyes.^3^ Another study showed no additional benefit to taping the eyelids of canine patients closed during GA compared with the application of lubrication alone.^4^

It is standard practice to use artificial ocular lubrication intraoperatively as prophylaxis against corneal surface disease in veterinary patients undergoing GA. Although protective measures such as eye lubrication or taping the eyelids closed have been proven to reduce the risk of corneal ulceration in both humans and dogs, there is a paucity of information related to GA-associated ocular injury in cats.^4,16^ This study’s primary objectives were to better define the prevalence for corneal injury in cats associated with GA while utilizing a standardized lubrication protocol and quantify changes in aqueous tear production in cats after GA. A secondary objective was to describe the risk factors associated with corneal injury after GA in cats. The ultimate goal of this study was to develop an ocular lubrication protocol to reduce negative outcomes for cats undergoing GA.

## Materials and methods

This study was approved by the University of Saskatchewan Animal Care Committee’s Animal Research Ethics Board (animal use protocol number 20230024) and complies with the Canadian Council on Animal Care guidelines, the University of Saskatchewan’s Animal Care and Use Procedures, and the Tri-Council memorandum of understanding﻿. Cats undergoing GA for non-ocular procedures at the Veterinary Medical Center (VMC) at the Western College of Veterinary Medicine between 1 May and 15 August 2023 were included. Cats with a previous history of eye disease, or eye disease at the time of the study, and patients in hospital receiving medication that may alter tear production (opioids, sedatives, etc) at the time of the study, were excluded. Inclusion in this study was voluntary with informed client consent.

An ophthalmic examination was completed by the same investigator (KH) under the supervision of a Diplomate of the American College of Veterinary Ophthalmologists-certified veterinary ophthalmologist or a second-year veterinary ophthalmology resident to confirm the absence of pre-existing ocular disease, including assessing tear production, intraocular pressure, fluorescein stain (FS) testing and slit lamp biomicroscopy. FS and a Schirmer tear test-1 (STT-1) were performed using an EagleVision Color Bar Schirmer Tear Test as a baseline value before the administration of anesthetic agents. As per Dawson and Sanchez,^3^ corneal erosion in this study was defined as superficial epithelial damage with no penetration into the basement membrane, which was seen as an obvious but patchy uptake of FS. By contrast, corneal ulceration was defined as stromal exposure with an obvious strong uptake of FS.^3^ There was no minimum STT-1 value required for enrolment in this study. Cats testing positive for FS uptake were closely monitored for signs of ocular discomfort but no additional treatment was prescribed, and owners were informed to monitor and contact us with any ocular signs that would warrant treatment.

The ocular lubrication protocol began at the time of anesthetic induction, and lubrication was repeated with a hydrophilic carbomer gel, Optixcare, every 15 mins until the patient was extubated as per hospital policy. At 1 h after extubation and the last application of eye lubricant, hourly assessments were performed, which involved a STT-1 and FS up to 4 h after extubation or until the time of discharge from hospital. Four hours after extubation was chosen as the end of the observation period based on the average time of discharge for surgical patients at the VMC. Anesthetic agents and the anesthesia protocol utilized remained under the direction of the anesthesiologist. Personnel involved were not masked to the study.

The following data were recorded for each patient: breed, sex, age (in months), weight (in kg), surgical procedure, surgical position of patient, anesthetic length from induction to extubation (mins), preoperative STT-1 value, postoperative STT-1 values (1, 2, 3 and 4 h after), preoperative FS result, postoperative FS result (1, 2, 3 and 4 h after), premedication drugs and doses, induction drugs and doses, maintenance inhalant used, postoperative drugs and doses, other intraoperative drugs and doses (if deemed necessary by the anesthesiologist), anesthetic reversal drug and doses (if deemed necessary by the anesthesiologist) and recovery grade given by the anesthesiologist. Medications used for anesthesia are described in Table 1.

**Table 1:** Anesthetic drugs administered

Preoperative sedative agents	Preoperative opioids	Induction agents	Maintenance agents	Postoperative NSAIDs	Postoperative opioids
– Dexmedetomidine (Dexdomitor; Zoetis)– Midazolam (Fresenius Kabi Canada)	– Hydromorphone (Sandoz)– Methadone (Comfortan; Dechra)– Butorphanol (Torbugesic; Zoetis)	– Alfaxalone (Alfaxan; Zoetis)– Propofol (Baxter)	– Isoflurane (Fresenius Kabi Canada)– Sevoflurane (Baxter)	– Meloxicam (Metacam; Boehringer Ingelheim)– Robenacoxib (Onsior; Elanco)	– Buprenorphine (Vetergesic; Ceva)– Fentanyl (Sandoz)– Morphine (Sandoz)

NSAIDs = non-steroidal anti-inflammatory drugs

Logistic regression was utilized to identify potential risk factors for corneal erosions to include in the multivariable logistic regression model building. Initial univariable regression was used to identify possible risk factors and factors that had a *P* value <0.2 were then used in logistic regression model building at the multivariable level. Potential risk factors were evaluated for collinearity. A *P* value <0.2 was used as a relaxed threshold to decide which variables warranted inclusion in the further multivariable model building. All data were recorded using commercial spreadsheet software (Excel Version 16.81; Microsoft) and the statistical analysis was performed with a commercial statistical software package (Stata 17; StataCorp).

To explore and describe tear production, separate STT-1 comparisons were performed for right and left eyes between each time point and compared with the baseline for the relevant eye. Normality was assessed both visually and using a Shapiro–Wilk test. A paired *t*-test was used to compare group means, and the threshold for significance was adjusted with a Bonferroni correction for multiple comparisons on the same animal (ie, two eyes and multiple times). Eight comparisons were made; therefore, the threshold for significance was 0.006 (0.05/8).

## Results

The current study population had limited differences in breed/facial conformation, age, procedure or anesthetic protocol, which led to a limited ability to identify risk factors for corneal injury and reduced aqueous tear production. Descriptive statistics of the population are defined in Table 2.

**Table 2:** Descriptive statistics of study population

	Fluorescein stain result		
	Negative	Positive	Total
Number of patients	28 (66.7)	14 (33.3)	42 (100.0)
Sex			
Male	9 (32.1)	6 (42.9)	15 (35.7)
Female	19 (67.9)	8 (57.1)	27 (64.3)
Eye affected			
Left eye	–	9 (64.3)	–
Right eye	–	5 (35.7)	–
Breed			
Domestic shorthair	26 (92.9)	14 (100.0)	40 (95.2)
Siamese	2 (7.1)	0 (0.0)	2 (4.8)
Procedure type			
Spay	17 (60.7)	8 (57.1)	25 (59.5)
Castration	7 (25.0)	6 (42.9)	13 (31.0)
Other	4 (14.3)	0 (0.0)	4 (9.5)
Surgical position			
Dorsal	19 (67.9)	8 (57.1)	27 (64.3)
Right lateral	1 (3.6)	1 (7.1)	2 (4.8)
Left lateral	7 (25.0)	5 (35.7)	12 (28.6)
Sternal	1 (3.6)	0 (0.0)	1 (2.4)
Premedication opioid			
Methadone	12 (42.9)	2 (14.3)	14 (33.3)
Hydromorphone	8 (28.6)	6 (42.9)	14 (33.3)
Butorphanol	8 (28.6)	6 (42.9)	14 (33.3)
Induction agent			
Alfaxalone	18 (64.3)	6 (42.9)	24 (57.1)
Propofol	2 (7.1)	2 (14.2)	4 (9.5)
None	8 (28.6)	6 (42.9)	14 (33.3)
Maintenance inhalant			
Isoflurane	19 (67.9)	9 (64.3)	28 (66.7)
Sevoflurane	1 (3.6)	0 (0.0)	1 (2.3)
None	8 (28.6)	5 (35.7)	13 (31.0)
Postoperative NSAIDs			
Meloxicam	19 (67.9)	11 (78.6)	30 (71.4)
Robenacoxib	5 (17.8)	3 (21.4)	8 (19.1)
None	4 (14.3)	0 (0.0)	4 (9.5)
Postoperative opioids			
Buprenorphine	24 (85.8)	14 (100.0)	38 (90.4)
Morphine	1 (3.6)	0 (0.0)	1 (2.4)
Fentanyl	2 (7.1)	0 (0.0)	2 (4.8)
None	1 (3.6)	0 (0.0)	1 (2.4)
Reversal administered			
Yes	10 (35.7)	8 (57.1)	18 (42.9)
No	18 (64.3)	6 (42.9)	24 (57.1)

Data are n (%)

NSAIDs = non-steroidal anti-inflammatory drugs

**Figure 1:**
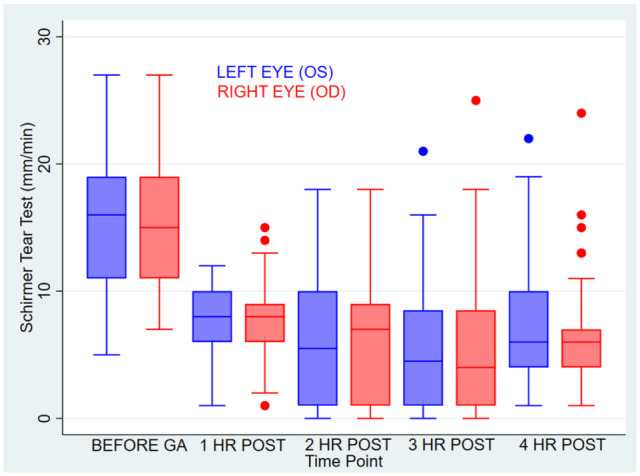
Trend of aqueous tear production before and after anesthesia. GA = general anesthesia

The mean pre-anesthesia STT-1 measurement was 15.6 mm/min in the left eye and 15.7 mm/min in the right eye (Figure 1). There was a statistically significant decrease in tear production for both eyes at all time points after GA (*P* <0.02).

Tear production was most severely decreased 2 and 3 h after extubation, as defined by the greatest mean difference in tear production (Table 3). No statistically significant clinical risk factors were found specific to tear production.

**Table 3:** Mean difference in aqueous tear production by eye

Time after extubation (h)	Left eye	Right eye
1	8.3 (6.3–10.4)	8.6 (6.7–10.3)
2	9.5 (7.4–11.6)	9.5 (7.2–11.8)
3	10.2 (7.9–12.5)	9.9 (7.7–12.3)
4	8.6 (6.3–10.9)	8.9 (6.9–10.9)

Data are mean (95% confidence interval) reported in mm/min

**Figure 2:**
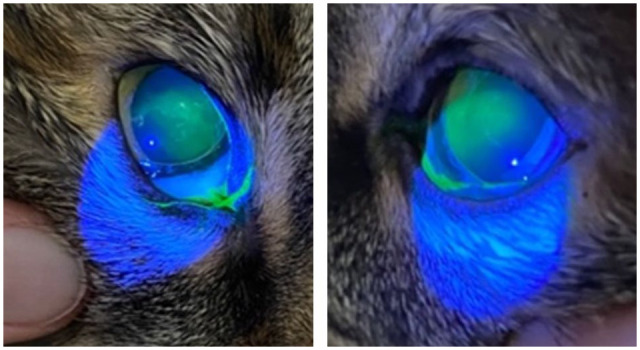
Example of a cat with bilateral corneal erosion after general anesthesia

No cats developed FS uptake consistent with superficial corneal ulceration. A total of 14 (33.3%) cats and 23 (27.4%) eyes developed faint, patchy corneal uptake of stain diffuse throughout the axial cornea consistent with epithelial erosion first identified 1 h after extubation (Figure 2). Of the 14 cats affected, nine were affected bilaterally and five unilaterally in the right eye. The FS result did not change between the hourly checks; all cats that were negative for stain uptake remained negative from the 1-h post-extubation check and continued to be negative until the period of observation ended (either 4 h after extubation or discharge from the hospital) and vice versa for cats that were positive for stain uptake. No patients re-presented with symptomatic corneal injury.

Preoperative opioid choice (methadone, butorphanol or hydromorphone) and the use of an induction agent were both correlated w﻿ith corneal injury at the univariable threshold of significance (*P* <0.2) and so were included in the multivariable model building. In contrast, preoperative opioid choice was not correlated with other predictive variables considered for association with corneal injury. After multivariable logistic regression, cats who received hydromorphone or butorphanol had 4.5 times higher odds of corneal erosion compared with cats who received methadone (*P* = 0.08).

There was a significant relationship between surgical position/corneal exposure and the development of corneal erosion (*P* = 0.003). All bilateral corneal erosions were identified in cats who were dorsally recumbent during surgery, while cats in lateral recumbency were more likely to have a single corneal erosion on the exposed eye as shown in Table 4.

**Table 4:** Descriptive statistics for the eye(s) affected by corneal erosion

	Both eyes	Right eye	Total
Total	9 (64.3)	5 (35.7)	14 (100)
Surgical position			
Dorsal recumbency	8 (88.9)	0 (0.0)	8 (57.1)
Left lateral recumbency	1 (11.1)	4 (80.0)	5 (35.7)
Right lateral recumbency	0 (0.0)	1 (20.0)	1 (7.1)

Data are n (%)

## Discussion

This study confirms that cats undergo a clinically significant reduction in aqueous tear production after GA, which is similar to findings in dogs.^3,4,6,8^ At 2 and 3 h after extubation, the majority of cats in this study were approaching STT-1 results of 0 mm/min. The data suggest that aqueous tear production may start to gradually recover by 4 h after extubation; however, as most cats in this study were discharged from hospital shortly after this time, we were unable to confirm when tear production returns to baseline. Herring et al^8^ described a significant decrease in aqueous tear production for up to 24 h after anesthesia in dogs, with a significant effect of duration of anesthesia on the post-anesthetic recovery of STT-1 values. Peche et al^9^ reported a reduction in aqueous tear production in cats for up to 18 h after GA. Longer term data collection to describe the timeline of post-anesthesia recovery of tear production is needed in cats to develop more specific postoperative lubrication protocols. However, based on the current information, frequent application of eye lubrication is necessary in the recovery period after GA in cats, and continuing artificial ocular lubrication for at least 24 h beyond the post-anesthesia recovery period is warranted.

There are many different types of ophthalmic lubricants that can be used to provide artificial lubrication during GA. Bedos et al^17^ compared different kinds of lubricants in healthy, non-anesthetized dogs and confirmed that ophthalmic ointments provide superior ocular lubrication to standard artificial tear solutions, and that lubricants containing hyaluronic acid had prolonged ocular surface contact time by reducing washout and enhancing water retention on the ocular surface. The present study utilized a carbomer-based lubricant without hyaluronic acid. Carbomer-based lubricants were not included in the study by Bedos et al;^17^ thus, it is not known how well these compare for contact time. In addition, the effects of anesthesia on artificial lubricant contact time and washout are unknown. Further investigations are needed to determine optimal lubrication protocols and formulations to reduce GA-related corneal injury in veterinary patients. The current hospital protocol for anesthetized patients is bilateral ocular lubrication every 15 mins by the anesthetist; however, further definition of an ideal lubrication protocol is needed for cats.

Corneal erosion occurred in 27.4% of eyes, which is higher than the range of 8–18.6% reported in dogs utilizing a similar ocular lubrication protocol, suggesting that cats may be at an increased risk of corneal injury after anesthesia compared with dogs.^3,4,18^ These results repeated in the present study emphasize that postoperative protection is essential to aid feline patients in the recovery period. The reason for this difference is unknown; however, it is possible that conformational differences, such as the more frontally located feline eye, may result in more potential for corneal exposure. Based on the axial location of GA-associated corneal injury in dogs, Ioannides et al^4^ suggested that the time of corneal injury is more likely to be the immediate pre- or postoperative period when the eye is central, rather than during GA when the canine eye is rolled ventromedially, allowing more inherent coverage and/or protection. The study by Ioannides et al describes the impact of GA on tear production in dogs without a surgical procedure (dogs undergoing advanced medical imaging while anesthetized).^4^ It may be important to further describe the impact of a procedure (surgical position/manipulation, pain, surgical grade lighting, active warming equipment, etc) in future studies in both dogs and cats, as they described an incidence of corneal erosion in 8% of eyes that is lower than the 10% and 18.6% described in other canine literature.^3,4,18^

A reduced blink-rate after anesthesia could contribute to corneal exposure and injury. There is currently no reported difference between pre- and post-anesthetic blink rate in cats. An adequate anesthetic plane requires a diminished ability to blink. The blink reflex and blink rate were not evaluated in our study; however, it should be investigated in future research to compare pre- and post-anesthetic blink rates in our veterinary patients. Frequent STT-1 measurements completed in our study may have contributed to corneal irritation; however, the corneal erosions were noted at the first time point after GA and did not change with increasing STT-1 readings throughout the observation period, suggesting an association with the GA event rather than being caused by the STT-1 strips themselves.

The pathophysiology of GA-associated corneal injury in cats is likely multifactorial and could include physical exposure or trauma during GA, reduced tear production, loss of protective blink reflexes, certain anesthetic medications as well as other unknown factors. In the present study, an association between surgical positioning and the eye affected by corneal erosion was identified. Cats placed in left lateral recumbency were more likely to have corneal injury to the right eye, which faced upwards, potentially leading to more corneal exposure. Similarly, all cats with bilateral corneal erosion were dorsally recumbent during surgery. This suggests the need for diligent lubrication, particularly of the upward facing eye and more exposed corneas. An additional method of protecting the cornea during anesthesia is physical closure of the eyelids throughout the procedure with tape.^4^ However, no difference in outcome was noted between lubrication alone and lubrication followed by taping the eyes closed in dogs undergoing anesthesia.^4^ Taping the eyes closed during anesthesia has not been investigated in cats but may be worthwhile based on what appears to be a higher prevalence of GA-associated corneal injury in this species. Taping the eyelids closed in cats could provide ocular protection when the anesthetist is not reliant on the eyes to assess anesthetic depth, such as procedures of the face and neck.

An important finding in our study was a higher risk of corneal erosion in cats who received hydromorphone or butorphanol compared with methadone in their anesthetic protocol. There is currently very little described in the literature comparing the effects that different opioids have on tear production or the corneal epithelium in cats. Methadone is a full opioid agonist with high opioid receptor binding (mu, kappa and delta subtype) and a N-methyl-D-aspartate (NMDA) antagonist.^19^ In contrast, butorphanol is a partial opioid agonist at the mu opioid receptor and a full agonist at the kappa opioid receptor, whereas hydromorphone is a full opioid agonist.^20,21^ Neither have NMDA receptor activity. Future hypotheses for research include comparing how the different receptor activity and the difference in an opioid’s ability to cross the blood–brain barrier can impact the feline cornea and tear production. Methadone might be more appropriately prescribed to patients undergoing ocular procedures or patients with a history of ocular disease to try and reduce GA-associated corneal injury. Further studies specifically comparing the effect of different opioids and alpha-2 agonist agents on tear production and blink rate could better inform anesthetic and sedation protocols in patients with known ocular conditions.

This study has some important limitations to acknowledge. The population of cats studied was small and uniform to a degree that did not allow for thorough study into isolated risk factors such as breed, weight, age and so on. For ethical reasons, we did not include a population of cats receiving no prophylactic ocular lubrication during GA; therefore, we could not compare the prevalence of ocular injury with a control. We were not able to control for time of day in the measurement of STT-1s; therefore, the daily rhythm of tear production may have influenced the results.^7^ In addition, we were unable to follow patients beyond 4 h after extubation as most were elective spay/castrate procedures that were discharged shortly after recovery from anesthesia; thus, we remain unsure as to how long it takes for the STT-1 to normalize after GA.

## Conclusions

Prophylactic eye lubrication is crucial in our feline patients undergoing GA, both intra- and postoperatively, to aid in recovery and comfort, and is an easy addition to any postoperative discharge protocol. Diligent ocular lubrication is warranted beyond extubation and for several hours into the postoperative recovery period, as tear production has not normalized within 4 h of extubation in feline surgical patients. Cats appear to be at a higher risk for corneal injury compared with dogs, which could lead to clinical corneal ulceration and other ocular sequelae when neglected. Longer term data collection to describe the timeline of post-anesthesia recovery of tear production is needed in cats to develop better postoperative lubrication protocols. The evaluation of larger numbers of animals under differing anesthetic protocols and surgical procedures would be beneficial in both canine and feline patients to isolate risk factors and describe the complicated pathophysiology of decreased aqueous tear production and corneal injury after GA.
